# miR-193b Modulates Resistance to Doxorubicin in Human Breast Cancer Cells by Downregulating MCL-1

**DOI:** 10.1155/2015/373574

**Published:** 2015-10-07

**Authors:** Jingpei Long, Zhiwei Ji, Kai Jiang, Zhaoyang Wang, Guanmin Meng

**Affiliations:** ^1^Department of Surgery, The Women's Hospital, School of Medicine, Zhejiang University, Hangzhou 310006, China; ^2^School of Electronics and Information Engineering, Tongji University, Shanghai 201804, China; ^3^Department of Clinical Laboratory, Tongde Hospital of Zhejiang Province, 234 Gucui Road, Hangzhou 310012, China; ^4^Department of Ophthalmology, Xinhua Hospital, School of Medicine, Jiaotong University, Shanghai 200092, China

## Abstract

MicroRNAs (miRNAs) family, which is involved in cancer development, proliferation, apoptosis, and drug resistance, is a group of noncoding RNAs that modulate the expression of oncogenes and antioncogenes. Doxorubicin is an active cytotoxic agent for breast cancer treatment, but the acquisition of doxorubicin resistance is a common and critical limitation to cancer therapy. The aim of this study was to investigate whether miR-193b mediated the resistance of breast cancer cells to doxorubicin by targeting myeloid cell leukemia-1 (MCL-1). In this study, we found that miR-193b levels were significantly lower in doxorubicin-resistant MCF-7 (MCF-7/DOXR) cells than in the parental MCF-7 cells. We observed that exogenous miR-193b significantly suppressed the ability of MCF-7/DOXR cells to resist doxorubicin. It demonstrated that miR-193b directly targeted MCL-1 3′-UTR (3′-Untranslated Regions). Further studies indicated that miR-193b sensitized MCF-7/DOXR cells to doxorubicin through a mechanism involving the downregulation of MCL-1. Together, our findings provide evidence that the modulation of miR-193b may represent a novel therapeutic target for the treatment of breast cancer.

## 1. Introduction

Breast cancer is the most common cancer in women worldwide, which is expected to account for 29% of all new cancer cases in 2012 [1]. Surgery combined with chemotherapy is the most effective strategy for breast cancer therapy currently. However, most of the treatments are unsuccessful due to the secondary recurrence, metastasis, and drug resistance [2]. For patients who especially suffered from advanced unresectable breast cancer, systemic therapy with chemotherapy drugs is a standard treatment strategy, although the problem of drug resistance has become serious [3]. Doxorubicin (DOX), an anthracycline drug, is widely used against a wide range of cancers, such as hematological malignancies, lung cancer, and breast cancer by directly intercalating the double-strand DNA and inhibiting DNA topoisomerase II [4, 5]. However, the major problems with doxorubicin treatment are cardiotoxicity and the induction of multidrug resistance [6, 7]. Therefore, enhanced anticancer therapy through the reversal of inducible DOX resistance would be desirable.

MicroRNAs (miRNAs) are small noncoding RNAs that negatively modulate gene expression via RNA binding at imperfect complementary sequences within the 3′UTR of the target mRNA, resulting in degradation or translational inhibition [8]. Recent studies indicate that miRNAs regulate cell growth, differentiation, and apoptosis [9]. Interestingly, some studies suggest that miRNAs are involved in tumor cell resistance to chemotherapeutic agents, such as doxorubicin [10]. However, the role of miR-193b in drug resistance of breast cancer cells remains unknown. We therefore compared the expression levels of miR-193b in the drug-resistant human breast cancer cell line MCF-7/DOXR, which was established by continuous exposure to doxorubicin, with its parental MCF-7 cell line. Furthermore, we investigated whether overexpression of miR-193b could increase the DOX sensitivity and determined the potential role of MCL-1 in miR-193b-mediated regulation of DOX resistance in human breast cancer cells.

## 2. Materials and Methods

### 2.1. Reagents

Antibodies against MCL-1 and *β*-actin were purchased from cell signaling (USA). Doxorubicin (DOX), 3-(4,5-dimethylthiazol-2-yl)-3,5-diphenyltetrazolium bromide (MTT), and Annexin V-FITC Apoptosis Detection Kit were obtained from Sigma-Aldrich (USA). miR-193b mimic and negative control oligonucleotide (NCO) were purchased from Genepharma Company (China). The sequences were as follows: miR-193b mimic, AACUGGCCCUCAAAGUCCCG CU; NCO, GCUCCCAACCCUUGGCCCAAGU.

### 2.2. Cell Lines and Culture

MCF-7 cells were obtained from American Type Culture Collection (ATCC, USA) and cultured in DMEM basic medium (Gibco, USA) with 10% fetal bovine serum (FBS, Gibco, USA) at 37°C in a humidified 5% CO_2_ incubator. MCF-7/DOXR (doxorubicin-resistant MCF-7) cell line was established by stepwise exposure of MCF-7 cells to increasing concentrations of doxorubicin. The cells were initially treated with doxorubicin at 0.2 *μ*g/mL for 2 months, and then the concentrations of doxorubicin were increased by 0.03 *μ*g/mL every month up to a final concentration of 0.5 *μ*g/mL. The MCF-7/DOXR cells were exposed to doxorubicin over a time period of 12 months. Doxorubicin was removed from medium 3 days before any experiments were run.

### 2.3. Quantitative Real-Time Polymerase Chain Reaction (RT-qPCR)

Total RNA from the MCF-7 or MCF-7/DOXR cells was extracted with Trizol reagent (Invitrogen, USA). The expression of miR-193b was assayed using stem-loop reverse transcription (RT) (Chen et al. [11]) followed by real-time PCR analysis. The RT-primer sequences are as follows: 5′-CTCAACTGGTGTCGTGGAGTCGGCAATTCAGTTGAGAGCGGGAC-3′, and the cDNA was synthesized using M-MLV Reverse Transcriptase (Invitrogen, USA). The qPCR primers were used as follows: MCL-1 forward 5′-CCAATGGGCAGGTCTGG-3′, MCL-1 reverse 5′-TACGCCGTCGCTGAAAA-3′; GAPDH forward 5′-GCATCCTGGGCTACACTG-3′, GAPDH reverse 5′-TGGTCGTTGAGGGCAAT-3′. qPCR was performed using a standard protocol from the SYBR Green PCR kit (Toyobo, Japan) on the Applied Biosystems 9700 system (USA). U6 or GAPDH was used as the internal control, and the relative level of miR-363 or MCL-1 expression was determined with the 2^−ΔΔCT^ method to calculate the fold change of the RNA expression [12].

### 2.4. Recombinant Plasmid Construction

The 3′-UTR sequences of MCL-1 (GenBank accession number: NM_001197320) containing the putative miR-193b binding site were amplified by PCR using the cDNA from MCF-7/DOXR cells as a template. Primers for MCL-1 3′-UTR were as follows: forward, 5′-TACTGTAAGTGCAATAGT-3′; reverse, 5′-TACCATCTTCACTAAATCT-3′. PCR products were cloned into the pMIR-REPORT miRNA Expression Reporter Vector System (pMIR, Life Technologies, USA). The mutant plasmid was created by mutating the seed regions of the miR-193b binding sites using site-directed mutagenesis kit (Takara, Japan). MCL-1 expression vector was established for the “rescue” experiment, where the open reading frame of MCL-1 was cloned into pcDNA3.1 (Invitrogen, USA). The recombinant plasmid was named pcDNA3.1-MCL-1.

### 2.5. Transient Transfection

Human miR-193b (50 nM), NCO (50 nM), pcDNA3.1 (2 *μ*g/mL), pMIR reporter plasmid (2 *μ*g/mL), and control Renilla luciferase pRL-TK vector (100 ng/mL, Promega, USA) were transfected into MCF-7/DOXR cells using Lipofectamine 2000 (Invitrogen, USA) when the cells were covered at approximately 80% confluence of the plate.

### 2.6. Luciferase Assay

MCF-7/DOXR cells were transfected with the MCL-1 3′UTR pMIR firefly luciferase reporter vector, control Renilla luciferase pRL-TK vector, and miR-193b mimic or NCO using Lipofectamine 2000 reagent. After 48 h transfection, cells were lysed, and assays were performed using the Dual-Luciferase Reporter Assay System kit (Promega, USA) according to the manufacturer's instructions. Results were represented as the ratio between the treatment groups and the negative control groups.

### 2.7. Drug Sensitivity Assay

Cells were seeded onto 96-well plates with 5 × 10^3^ per well in growth medium and incubated for 12 h and then treated with doxorubicin at concentrations that ranged from 0.05 to 1.2 *μ*g/mL for MCF-7 and from 0.8 to 16 *μ*g/mL for MCF-7/DOXR, respectively. 48 h after doxorubicin treatment, MTT (20 mL, 5 mg/mL) was added to each well and incubated for 4 h. Medium was then aspirated and 100 *μ*L DMSO was added. Absorbance was read at OD 570/655 nm using 680 Microplate Reader (Bio-Rad, USA). Doxorubicin concentrations leading to 50% cell death (IC_50_) were determined by a MTT-dependent cell viability assay.

### 2.8. Western Blot Analysis

Cells were lysed in RIPA lysis buffer (Cell Signaling, USA). Protein concentrations were measured using a BCA Protein Assay kit (Pierce, USA) according to the manufacturer's instructions. Following electrophoresis on 12.5% SDS-PAGE, protein samples were transferred to a PVDF membrane (Millipore, USA). After incubating with anti-MCL-1 and anti-*β*-actin primary antibodies, the membranes were then incubated with horseradish peroxidase-conjugated secondary antibodies (Cell Signaling, USA). The protein levels were analyzed by enhanced chemiluminescence detection kit (Pierce, USA). *β*-actin was used as loading control.

### 2.9. Assessment of Apoptosis

Cells were transfected with mentioned RNA/DNA. After 24 h incubation, doxorubicin was then added to the cell medium at a concentration of 1.2 *μ*g/mL. After another 24 h incubation, cells were collected and assayed with an Annexin V-FITC Apoptosis Detection Kit on the flow cytometry (Becton Dickinson, USA).

### 2.10. Statistical Analysis

In all the experiments, data are expressed as mean ± SE and derived from at least three independent experiments. All the statistical analyses were performed using SPSS12.0 software (USA). The difference between mean values was analyzed with Student's *t*-test. *p* values of <0.05 were considered to be statistically significant.

## 3. Results

### 3.1. Downregulation of miR-193b and Upregulation of MCL-1 in Breast Doxorubicin-Resistant Cells

**Figure 1:**
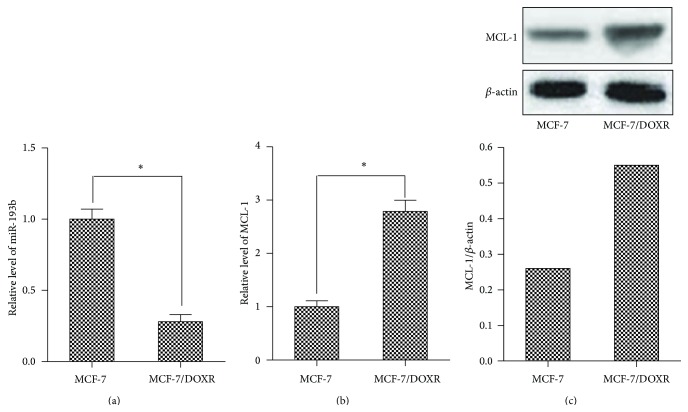
Downregulation of miR-193b and overexpression of MCL-1 in MCF-7/DOXR cells. (a) The miR-193b expression in MCF-7 and MCF-7/DOXR cells was detected by qPCR. ^*∗*^ *p* < 0.05. (b) qPCR analysis for MCL-1 mRNA expression levels in MCF-7 and MCF-7/DOXR cells. ^*∗*^ *p* < 0.05. (c) Western blot analysis for MCL-1 protein levels in MCF-7 and MCF-7/DOXR cells.

To investigate the relationship between miR-193b expression and doxorubicin resistance, we established a doxorubicin-resistant MCF-7 cell line (MCF-7/DOXR) by continuous exposure of MCF-7 cells to doxorubicin. As shown in Figure 1(a), the levels of miR-193b were significantly lower in MCF-7/DOXR cell line than in its parental MCF-7 cell line. We then subsequently performed qPCR and western blot to detect the expression of MCL-1. As expected, both the mRNA and protein expression levels of MCL-1 in MCF-7/DOXR cells were higher than that in its parental MCF-7 cells (Figures 1(b) and 1(c)). These data indicate that the downregulation of miR-193b and upregulation of MCL-1 may be related to doxorubicin resistance.

### 3.2. Transfection of miR-193b Sensitized MCF-7/DOXR Cells to Doxorubicin

**Figure 2:**
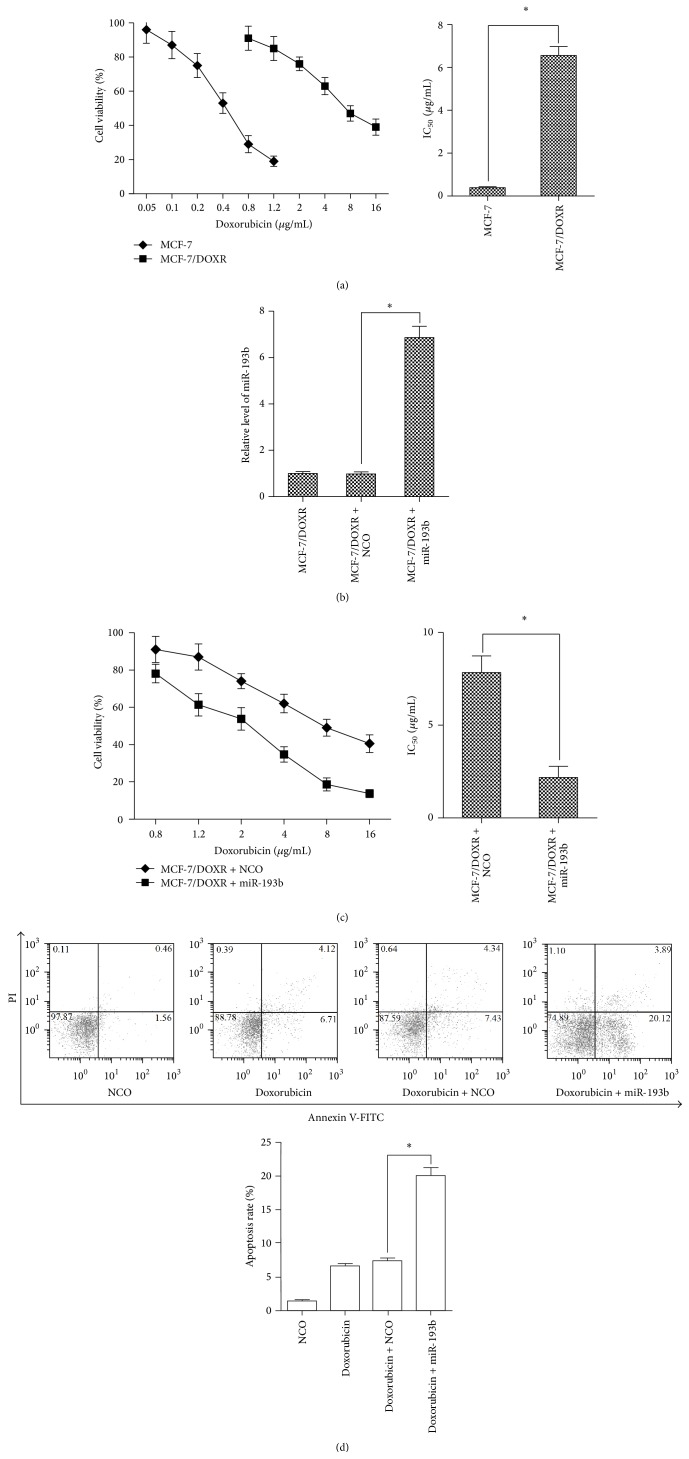
Transfection of miR-193b sensitized MCF-7/DOXR cells to doxorubicin. (a) MCF-7 and MCF-7/DOXR cells were treated with different concentrations of doxorubicin, and cell viability was assayed by using MTT. The IC_50_ was determined according to the survival curves, which showed the MCF-7/DOXR cell line got a 17.0-fold acquired resistance to doxorubicin compared to the MCF-7 cell line (6.560 ± 0.412 versus 0.386 ± 0.051 *μ*g/mL, ^*∗*^ *p* < 0.05). (b) MCF-7/DOXR cells were seeded in 6-well plates and transfected with miR-193b mimic or NCO, and the miR-193b expression level was assayed by qPCR. ^*∗*^ *p* < 0.05 versus NCO group. (c) MCF-7/DOXR cells were treated with different concentrations of doxorubicin plus miR-193b or NCO, and cell viability was assayed by using MTT. The IC_50_ was significantly lower in MCF-7/DOXR cells transfected with miR-193b as compared to the cells transfected with NCO, ^*∗*^ *p* < 0.05. (d) MCF-7/DOXR cells were treated with 1.2 *μ*g/mL doxorubicin plus miR-193b or NCO, and cell apoptosis was detected by Annexin V/PI staining. Data are expressed as the means ± SE (*n* = 3). ^*∗*^ *p* < 0.05.

Doxorubicin markedly inhibited the viability of MCF-7 in a dose-dependent manner. However, we found approximately 17.0-fold increase of IC_50_ for the MCF-7/DOXR cells in the presence of doxorubicin (IC_50_ = 6.56 *μ*g/mL), as compared to the parental MCF-7 cells (IC_50_ = 0.39 *μ*g/mL), indicating that the MCF-7/DOXR cells were resistant to doxorubicin (Figure 2(a)). To investigate whether miR-193b modulated chemosensitivity in breast cancer, we transfected 50 nM miR-193b mimic or NCO into MCF-7/DOXR cells.  Figure 2(b) showed that miR-193b levels were effectively elevated by transfection with the mimics in the MCF-7/DOXR cells. We observed that the MCF-7/DOXR cells transfected with miR-193b exhibited significantly enhanced sensitivity to doxorubicin comparing with those transfected with NCO, with IC_50_ values of 2.180 ± 0.602 and 7.840 ± 0.901 *μ*g/mL, respectively (Figure 2(c)). To further investigate the apoptosis-inducing effect of doxorubicin plus miR-193b in MCF-7/DOXR cells, Annexin V/PI staining for apoptosis was performed. As shown in Figure 2(d), miR-193b enhanced apoptosis in MCF-7/DOXR cells during the doxorubicin treatment. These results suggest that miR-193b increases the sensitivity of breast cancer cells to doxorubicin.

### 3.3. miR-193b Directly Targets MCL-1 and Suppresses Its Expression in MCF-7/DOXR Cells

**Figure 3:**
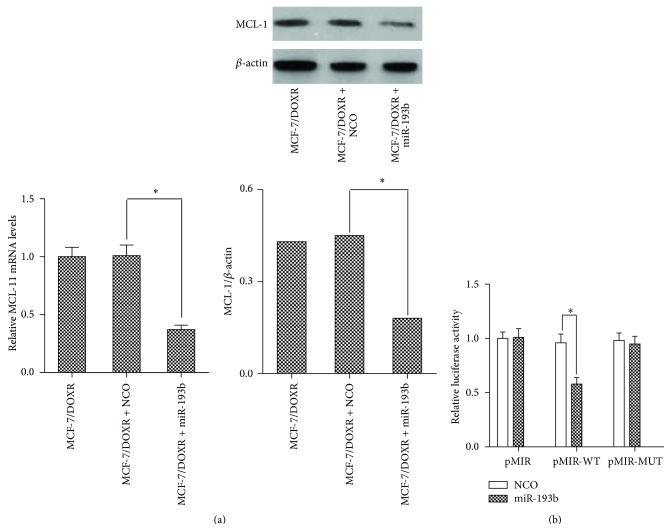
miR-193b directly targets MCL-1 and suppresses its expression in MCF-7/DOXR cells. (a) MCF-7/DOXR cells were transfected with miR-193b mimic or NCO incubating for 24 h, and then the mRNA expression and protein expression of MCL-1 were detected by qPCR analysis and western blot, respectively, ^*∗*^ *p* < 0.05. (b) Effect of miR-193b on MCL-1 was assessed with the dual-luciferase reporter system. The miR-193b mimic, pRL-TK, and pMIR reporter containing wild- or mutant-type 3′-UTR of MCL-1 gene were cotransfected into the MCF-7/DOXR cells, ^*∗*^ *p* < 0.05.

MCL-1 has been reported as a potential target gene of miR-193b [13]. To further explore the downstream mechanism through which miR-193b modulates doxorubicin resistance, we transfected the MCF-7/DOXR cells with miR-193b mimic or NCO. After 24 h incubation, a significant decrease in both mRNA and protein expression levels of MCL-1 was observed in MCF-7/DOXR cells transfected with miR-193b (Figure 3(a)). Furthermore, we cotransfected miR-193b mimic or NCO together with the pMIR-reporter luciferase plasmid containing 3′UTR of MCL-1 into the MCF-7/DOXR cells. As shown in Figure 3(b), miR-193b significantly suppressed the luciferase activity of the pMIR reporter with wild-type 3′-UTR of MCL-1, whereas the mutant MCL-1 3′UTR or empty pMIR-luciferase activity remained unchanged in cells transfected with miR-193b. These results indicate that MCL-1 is negatively regulated by miR-193b as its direct target gene in MCF-7/DOXR cells.

### 3.4. miR-193b Sensitized MCF-7/DOXR Cells to Doxorubicin through the Downregulation of MCL-1

**Figure 4:**
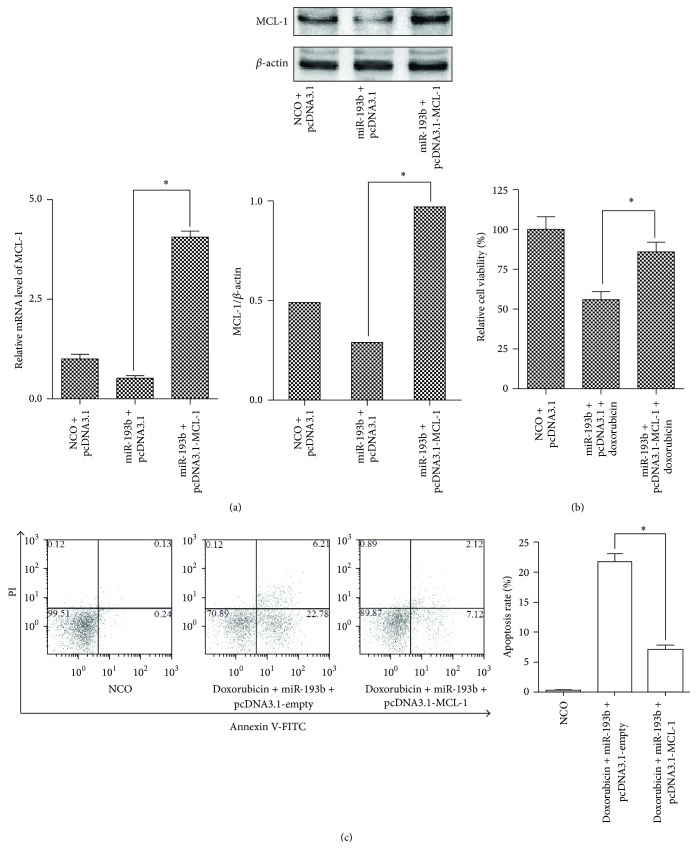
miR-193b sensitized MCF-7/DOXR cells to doxorubicin depending on the downregulation of MCL-1. (a) MCF-7/DOXR cells were cotransfected with miR-193b or NCO plus empty pcDNA3.1 or pcDNA3.1-MCL-1. MCL-1 mRNA and protein expression levels were assayed by qPCR and western blot, respectively, ^*∗*^ *p* < 0.05. (b) MCL-1 expression vector rescued the MCF-7/DOXR cells treated with doxorubicin (1.2 *μ*g/mL) plus miR-193b (50 nM), suggesting miR-193b modulates doxorubicin resistance through targeting MCL-1. (c) Enforced expression of MCL-1 decreased the apoptosis induced by doxorubicin plus miR-193b. Apoptosis was measured using Annexin V/PI staining by flow cytometry. Data are expressed as the means ± SE (*n* = 3). ^*∗*^ *p* < 0.05.

To investigate the role of MCL-1 in miR-193b regulated doxorubicin resistance, we rescued the doxorubicin- and miR-193b-treated MCF-7/DOXR cells with MCL-1 expression vector. We observed that the transfection of pcDNA3.1-MCL-1 totally reversed the downregulation of MCL-1 by miR-193b (Figure 4(a)). More importantly, the overexpression of MCL-1 significantly reversed the viability of the MCF-7/DOXR cells treated with miR-193b and doxorubicin (Figure 4(b)). Furthermore, we also examined the effect of MCL-1 overexpression on apoptotic cell death in MCF-7/DOXR cells treated with doxorubicin and miR-193b. The flow cytometry analysis showed that the number of apoptotic cells in pcDNA3.1-MCL-1 group was dramatically less than that in the empty pcDNA3.1 group (Figure 4(c)). Taken together, MCL-1 is a potential target through which miR-193b modulates doxorubicin resistance in human breast cancer.

## 4. Discussion

Although advances in both diagnosis and treatment for malignancy have contributed to the improvement of prognosis [14, 15], current available therapeutic options are very limited for patients with advanced breast cancer while the tumor tissues have developed beyond curative surgery. Chemotherapy plays an important role in the treatment of advanced breast cancer. However, acquired chemoresistance is becoming a major challenge for the patients [16]. In our study, the MCF-7/DOXR cell line was proximately 17.0 times more resistant to doxorubicin as compared with its parental MCF-7 cell line, suggesting that the continuous exposition to doxorubicin enabled breast cancer cells to acquire drug resistance. Therefore, there is an urgent need to identify the key molecules involved in breast cancer chemoresistance in order to develop novel strategies for breast cancer.

Recent studies have shown that some specific miRNAs can modulate drug resistance. Recent studies demonstrate that miR-30c, one of the well-known tumor suppressor miRNAs that promote cell death and inhibit tumor invasion, plays an important role in reversing chemotherapy resistance by regulating TWF1 and interleukin-11 [17, 18]. miR-200c expression is reported to be downregulated in multidrug-resistant MCF-7 cells as compared to the parental MCF-7 cells, while upregulation of miR-200c by its mimics could enhance the chemosensitivity to epirubicin in breast cancer cells [19]. Similar to the aforementioned findings, in the present study, we found that the expression of miR-193b was lower in MCF-7/DOXR cells comparing with its parental MCF-7 cells. More importantly, transfection of MCF-7/DOXR cells with the exogenous miR-193b significantly decreased doxorubicin resistance. Furthermore, our study provided new insight into the molecular basis of doxorubicin resistance; that is, miR-193b sensitizes breast cancer cells to doxorubicin by targeting myeloid cell leukemia-1 (MCL-1).

MCL-1 is an important member of antiapoptotic BCL-2 family proteins, which is overexpressed in many human cancer cells associated with poor prognosis [20, 21]. MCL-1 is known as a tumor survival factor via suppressing the apoptosis by inhibiting the normal function of proapoptotic Bcl-2 family proteins such as Noxa [22]. Moreover, MCL-1 is reported to mediate chemotherapy resistance in multiple cancers [23, 24]. In this study, we found that MCL-1 was significantly overexpressed in MCF-7/DOXR cells, suggesting that the MCL-1 might be essential for doxorubicin resistance in breast cancer. Further results showed that MCL-1 was directly regulated by miR-193b, which is in accordance with the prior finding in melanoma [13]. Interestingly there was a negative correlation between the expression levels of miR-193b and MCL-1 in MCF-7/DOXR cells. More importantly, we found doxorubicin-induced apoptosis was inhibited in MCF-7/DOXR cells cotransfected with MCL-1 expression vector and miR-193b mimic, indicating that MCL-1 plays a pivotal role in mediating miR-193b-modulated doxorubicin resistance in human breast cancer.

## 5. Conclusion

We conclude that the miR-193b modulates resistance to doxorubicin in human breast cancer cells by downregulating MCL-1. Aberrant regulation of apoptosis commonly plays a key role in acquired chemotherapy resistance for cancer cells, which is largely mediated by the Bcl-2 family proteins [25]. So the present study may provide a novel individualized treatment strategy for chemotherapy-resistant tumors by miR-193b-MCL-1-apoptosis pathway.
